# Editorial: Plant-microbe interactions in forest ecosystems

**DOI:** 10.3389/fpls.2022.1111566

**Published:** 2022-12-14

**Authors:** Luciano Kayser Vargas

**Affiliations:** Laboratório de Química Agrícola, Departamento de Diagnóstico e Pesquisa Agropecuária (DDPA), Secretaria Estadual da Agricultura, Pecuária e Desenvolvimento Rural, Porto Alegre, Brazil

**Keywords:** forest, mycorrhizal fungi (MF), microbial diversity, nitrogen fixation, cyanobacteria, phytopathogenic fungi

Forests, both natural and planted, are complex and dynamic ecosystems in which a multitude of below-ground interactions between plants and soil microbes occur. Positive and negative plant-microbe interactions impact the productivity and sustainability of forests. In agricultural soils, plant-microbe interactions are being studied more intensely, resulting in the development of technological solutions that are already being made available in agriculture. Plant-microbial interactions in forests are far less studied and represent a production bottleneck, both for companies and farmers who commercially explore planted forests and for the sustainable utilization or recovery of natural forests.

The sustainability of our planet is highly dependent on forest ecosystems. From an economic point of view, they directly contribute to our natural and processed products, such as wood and cellulose. From an environmental point of view, they play a role in the preservation of biodiversity, acting as a refuge for native flora and fauna. In addition, they promote carbon sequestration and preserve the quality of soil, air, and water. In this context, understanding diversity and microbial activity under different methods of forest management, identifying positive and negative interactions between microbes and forest species, and using these microorganisms as technological tools can make forest production more efficient and environmentally friendly, from seed germination or the rooting of cuttings to produce seedlings up until the end of the tree’s life cycle.

Temperate and boreal forests are usually dominated by a few tree species, while presenting below-ground diverse ectomycorrhizal (ECM) fungal communities that interconnect plants by mycelial networks and regulate nutrient and carbon cycles, influencing soil structure and ecosystem functionality (Van der Heijden et al., 2015). The abundant-center hypothesis, which postulates that species abundance peaks in the center of its distributional range and declines toward its edge, was tested by Wang and Han in ectomycorrhizal (ECM) fungal communities associated with *Picea crassifolia*, an endemic coniferous species widely distributed in North-west China. The authors analyze the taxonomic richness and relative abundance of ECM fungi in four main distribution areas, from the center to the edges. ECM fungal richness and biodiversity are highest at the central points and lower at the peripheral sites, indicating that ECM fungal species richness is consistent with the abundant-center hypothesis, although the relative abundances of individual fungal genera shifts inconsistently across the plant’s distribution.

Tropical forests are, however, more diverse in vegetation structure and composition (Rajpar, 2018), and the presence of arbuscular mycorrhizal fungi (AMF) is a key factor in the maintenance of tree diversity (Lovelock et al., 2003). Conversely, plant diversity also affects AMF diversity, as shown by Edy et al. The authors evaluate how the conversion of tropical lowland rain forests into rubber tree and oil palm plantations affects the associated mycorrhizal fungi. The authors call attention to the loss of biological heritage since some vanished AMF species may not be recovered, with unpredictable consequences for long-term ecosystem stability.

Contrary to mycorrhizal fungi, phytopathogenic fungi interact negatively with plants, endangering forest sustainability. In this context, the review of Amaral et al. discusses the main advances in the knowledge of pine pitch canker, one of the most prevalent diseases affecting conifers, caused by the fungus *Fusarium circinatum*. Chai et al., in turn, demonstrate the role of fungi, along with actinomycetes in a lesser-known process, soil water repellency (SWR). The authors verify that the lower microbial diversity of *Pinus* forest soil, compared with *Robinia* and *Hippophae*, results in higher polar wax content and, as a consequence, higher SWR.

The relationship between forest plant composition and microbial diversity is also evidenced by Lyu et al. The researchers constructed forest gaps of three different sizes in weeping cypress plantations and evaluate plant diversity, physicochemical properties of the soil, and bacterial diversity, compared with plots without any gaps. Larger gaps significantly increase plant diversity in the understory layer, which is the most important environmental factor driving the composition and diversity of bacterial communities.

Finally, Arróniz-Crespo et al. unveil new pathways for cyanobacterial-fixed N_2_ to enter the boreal forest ecosystem. As pointed out by the authors, biological N_2_ fixation in feather mosses is one of the largest inputs of nitrogen to boreal forest ecosystems. It was assumed that fixed N_2_ was sequestered into moss tissue and only released after decomposition. However, NanoSIMS images reveal that newly fixed N_2_ is incorporated not only into moss tissues but also into associated bacteria, fungi, and microalgae. That is the first empirical evidence that N_2_ fixed by cyanobacteria can enter the boreal forest ecosystem through the feather-mosses microbiome.

This Research Topic brings together articles that address different aspects of the relationship between microorganisms and forests. The articles cover completely different biomes and climates, as well as diverse and often neglected components of the microbiota associated with different forest species. We invite readers to appreciate in detail each of the articles that make up this collection, in the hope that they can stimulate new research on the subject.

## Author contributions

The author confirms being the sole contributor of this work and has approved it for publication.
